# A Delphi study for setting up tobacco research and practice network in India

**DOI:** 10.1186/s12971-016-0067-x

**Published:** 2016-02-02

**Authors:** Divya Persai, Rajmohan Panda, Ravi Kumar, Andy Mc.Ewen

**Affiliations:** Public Health Foundation of India, Delhi NCR, Gurgaon, India; Principal Research Associate, Epidemiology & Public Health, Institute of Epidemiology & Health, Faculty of Pop Health Sciences, University of London, London, UK

## Abstract

**Background:**

There are key gaps in the production and dissemination of evidence-based tobacco control research in developing countries. In India, limited research has been made to address and understand the function, constitution, thematic areas of research of a research and practice network in tobacco control. This study aimed to identify priority areas that were agreed by stakeholders for building tobacco research and practice network in India.

**Methods:**

This study used the well-established Delphi survey, which involves asking experts a recurring progression of questions through a series of questionnaires. The study was conducted in two rounds in the year 2013–14. Experts working in tobacco control participated in the study. In Round II, respondents rated agreement using a five-point Likert scale. Interquartile Range (IQR) was used to calculate the strength of the consensus.

**Results:**

Experts expressed strong consensus on tobacco cessation and economic research as a focus areas for tobacco research network in India. Lack of funding was stated as a barrier impeding formation of tobacco research network in India by majority of respondents. A strong consensus was achieved on the fact that network can be sustained financially by Government funds (IQR = 1). Information sharing and capacity building of young researchers were the two major benefits as stated by respondents which achieved strong consensus.

**Conclusions:**

This study produced the first national stakeholder-informed priority area for developing tobacco research and practice network in India. The consensus priorities highlight the most important and urgent needs in developing research and practice network in tobacco control.

## Background

Tobacco use is the single most important cause of preventable morbidity and premature mortality [1]. In India over a dozen distinct forms of tobacco are consumed by millions of people [2]. In comparison to the huge diversity of the problem, tobacco research is still in its nascent stages. In Low and Middle Income Country (LMIC) such as India, few people work full time on tobacco control. Even fewer are involved in tobacco control research that is relevant for the populations. A plethora of research conducted in LMICs has yet to be summarized and disseminated and important combinations and linkages have yet to be made within the voluminous evidence produced by researchers [3]. Studies have suggested that the barriers to research in tobacco control in India include lack of data standardization and weak communication networks [4]. There are also key gaps in the production and dissemination of evidence-based tobacco control research. Evidence-informed policies and programs can easily be impeded when there is insufficient capacity to produce new and relevant research to meet the information need of decision makers in timely manner [5]. On the other hand failure to translate evidence into practice threatens to undermine much of the excellent tobacco control work undertaken in many low- and middle-income countries [6]. In addition, linkages within selected expert groups have limited the work in consensus building on contentious issues in tobacco control. Overall a collaborative and cohesive approach to respond to the problem has not been the characteristic of research in tobacco control. One of the reasons for such disconnect amongst researchers could be a lack of a national tobacco research network in the country where researchers can connect with each other and share ideas, protocols, and findings. While many of the electronic networks have contributed positively to the discussion of tobacco control, they are not universally accessible in the developing world [7].

Research of different types can contribute to more effective choice and implementation of tobacco control activities but resources are limited and expertise in this field is scarce. There is a case for concentrating research resources on investigating targeted problems and potential solutions in tobacco control. If researchers and funders were to concentrate efforts and resources, then which topics should be the focus and which criteria should be used to set priorities? Should choices be made on the likelihood of the question being answerable by current methods? We believe that the research platform would only work if the framework of such a platform is developed by inputs from researchers throughout the country. In India, limited research has been made to address and understand the function, constitution, thematic areas of research and establishment of a practice network in tobacco control. The present study aimed to assess the perceptions of varied stakeholders working in tobacco control to guide the formulation of the Tobacco Research and Practice Network in India. The purpose of this paper is to present a new conceptual framework for developing tobacco research and practice network in India.

## Methods

This study used the Delphi survey, which involves asking questions through a series of questionnaires. The Delphi method has been used to develop research priorities in many areas of health. We selected it because it is known to be effective when (1) consensus is sought in an area where none previously existed, (2) the research problem does not lend itself to precise analytical approaches but can be illuminated by subjective collective judgments, (3) the study respondents have diverse backgrounds in their experience and expertise and therefore consensus cannot easily be reached [8, 9].

### Questionnaire and methodology

In this study a two-round survey strategy was used. The first step consists of literature review and Round I survey to identify emerging topic areas and themes. All topic areas identified in both the literature review and Round I survey were included in Round II of the Delphi survey. The Delphi survey was conducted online in two rounds [November-December 2013 and July – November 2014 respectively]. The questionnaire for Round I (online survey) included 11 items (Table 1). The questionnaire for the first round was pretested and the initial invitation to participate in the survey was accompanied by a brief project description.

**Table 1:** Survey tools for Round I & II

Survey tool for Round I
What are the main factors holding back the formation of Tobacco control Research and practice Network (TRNI) in India?
What should be the priority of the TRNI?
What should be the focus of TRNI?
How TRNI can aid in implementation of tobacco control policies and program in the country?
How the quality of good tobacco control research and practice network can be assured?
What are the activities which can be undertaken in the TRNI?
How TRNI can financially sustain?
How TRNI can help in your research and practice?
Survey tool for Round II
Below, is a list of the focus areas identified for tobacco research and practice network. Please rate each of the areas in terms of its value in developing a Tobacco Research and Practice Network in India.
How can Tobacco Research and Practice Network influence evidence-based implementation of tobacco control policies and programs?
What are the main factors holding back research dissemination & knowledge sharing in research pertaining to tobacco control in India?
How can the TRNI address such issues?
According to you, who should be the part of tobacco research network in India?
What activities can enhance information exchange between researchers in the Tobacco Research and Practice Network
How TRNI would benefit researchers?

**Table 2:** Three-stage process for establishing consensus on research priorities

Stage 1	Stage 2	Stage3
Topic identification	Stakeholder survey	Priority consensus
Topics identified through literature review	Survey instrument was developed and refined as follows: − Online survey pilot − Online survey data were collected from respondents (*n* = 36; 60 % response rate)	Consensus formed by expert panel (Delphi process) (*n* = 10) − Expert panel completed online survey − Consensus declared by facilitator and group
Result	Result	Result
Topics were incorporated into survey instrument	Stakeholder survey data provided foundation for consensus formation	Research priorities finalized and disseminated

After analyzing the results of the first round, one of the facilitators provided an anonymous summary of the results. In the analysis between the first and the second round, full-text suggestions were structured and grouped into categories from which new items were developed and incorporated into the second-round questionnaire. Following the second and last round of the survey, the experts were provided a summary of results. This final feedback closed the exercise (Table 2). A five-point, single, ordinal, Likert-type scale was used to assess the opinion on each item. Following Delphi categorization, responses were classified in three regions: (1–2) = “disagree”; (3) = “neither agree nor disagree”; (4–5) = “agreement”. The survey also offered the possibility of adding individual explanatory observations to every answer. Highlights of survey to set priorities for TRNI are:

1. Research
  ▪ Focus of tobacco research network in India
  ▪ Factors holding back research dissemination & knowledge sharing in research pertaining to tobacco control
  ▪ Evidence-based implementation of tobacco control policies and programs
2. Organizational Structure & Funding
  ▪ Members of Tobacco research network in India
  ▪ Financial sustainability of tobacco research network in India
3. Partnerships & Networking
  ▪ Information sharing among members of TRNI
  ▪ Benefits of Research Network

### Respondents

Researchers and practitioners were sampled according to their expertise. In the first round, the survey instrument was sent to 60 researchers working in tobacco control across different regions of the country.

As the Delphi technique is a qualitative method, the samples needed to be comparable in terms of homogeneity but not in terms of representativeness. Nevertheless, the Delphi technique is valid, in terms of its effect on outcomes, irrespective of sample size [10]. A rigorous procedure has been followed to ensure the identification of relevant experts and gave them the opportunity to participate in the study. Characteristics of the final sampling frame were reviewed to assure that it included policy makers, researchers, academia and Non-government organizations. Potential respondents for the survey were identified as those who had multiple publications in tobacco-related research or who have expertise in tobacco control.

### Analysis

Based on the results of Round I, research areas were identified and mutually exclusive research questions were framed. Analysis of surveys in rounds II used descriptive statistics. Interquartile ranges (IQRs) were calculated for the panel responses to each question. The “level of agreement or disagreement” achieved was measured according to the following criteria: Consensus on a questionnaire item was considered “strong” when at least 75 % of respondents reached an agreement. “Moderate” consensus required 60 % to 74 % of respondents to agree on individual items of the questionnaire. Absence of consensus was determined when less than 60 % of respondents agreed on the individual items. Interquartile range (IQR) is used to calculate the strength of the consensus. IQR is the absolute value of the difference between the 75th and 25th percentiles, with smaller values indicating higher degrees of consensus. Interquartile range of 0 specifies a strong group consensus and 2 indicates dispersed responses [11, 12]. Statistical analyses were performed using SPSS Version 17.

The study was approved by the institutional ethical committee. The respondents provided their written informed consent to participate in the study. The institutional ethics committee approved the consent procedure.

## Results

### Participation, attrition, demographic data

Of the 60 experts whom we attempted to contact, 36 experts agreed to participate in the first round of the survey. A total of 10 experts participated in the second round. The average years of experience of respondents in tobacco control were 10 years. Professional backgrounds of the experts in both rounds were in national and international NGOs (31 %), Government (31 %), research organizations (11 %), and medical institutes (27 %).

Detailed results of each item (interquartile range and consensus result) are depicted in Table 3. Overall, consensus was reached in 26 items 18 of them (69 %) were in terms of agreement and the remaining 8 items (31 %) in terms of disagreement with the assertion presented.

**Table 3:** Experts consensus in Delphi study

Strong consensus	Consensus (agreement in %)	Inter quartile range
Focus of TRNI	>75 %	1
Tobacco cessation	>75 %	1
Economic research	>75 %	1
Implementation of tobacco control policy		
Sharing best practices	>75 %	1
Advocacy	>75 %	1
Financial sustainability		
Funds from government	>75 %	1
Network members		
Government	>75 %	1
NGOs	>75 %	1
Academia	>75 %	0.25
Factors holding back research dissemination		
Lack of funds		0
Benefits of the network		
Information sharing	>75 %	1
Technical support	>75 %	1
Moderate consensus		
Focus of TRNI		
Disease research	60–74 %	1
Implementation of tobacco control policy		
Consensus building	60–74 %	2
Establishing standard protocols	60–74 %	1
Factors holding back research		
Lack of technical expertise	60–74 %	2
Financial sustainability		
Funds from International organizations	60–74 %	1
Involvement of private sector and pharmaceutical companies		
Private sector should be involved with caution	60–74 %	
Benefits of the network		
Evidence-based policy implementation	60–74 %	
No consensus		
Factors holding back research		
Lack of emphasis on tobacco control	<60 %	2
Lack of platform/forum	<60 %	2
Financial sustainability		
Nominal membership fees	<60 %	3
Involvement of private sector and pharmaceutical companies		
Private sector can be in involved in the network	<60 %	2
Pharmaceutical companies should be involved with caution	<60 %	2
Private sector and pharmaceutical companies should not be involved in the network	<60 %	2
Benefits of the network		
Consensus building	<60 %	2

#### Barriers in formation of tobacco research network in India

In the first round, more than two-third of the respondents stated that lack of emphasis on tobacco-related research (77 %) and lack of collaboration between researchers and research organizations (62 %) are the main barriers in formation of tobacco research and practice network in India. Respondents stated that other factors which impede formation of a network are lack of funding (39 %) and resistance among researchers in sharing data (19 %). In the second round, experts had strong consensus on lack of funding as a barrier impeding formation of tobacco research network in India (IQR = 0). However, no consensus was achieved on lack of emphasis on tobacco-related research in the Round II of Delphi survey.

#### Focus of research and practice network in tobacco control

Experts were asked about their opinion on focus of the research and practice network. In Round I, all the respondents agreed on the fact that the focus of research and practice network should be on surveillance and legislation. More than two-third of the respondents agreed that the focus should be on tobacco cessation, diseases research and economics and taxation-related research. However, in Round II experts expressed strong consensus on tobacco cessation and economic research (IQR = 1) as a priority area of focus for tobacco research network in India.

#### Network’s role in implementation of tobacco control policies and program

**Fig 1:**
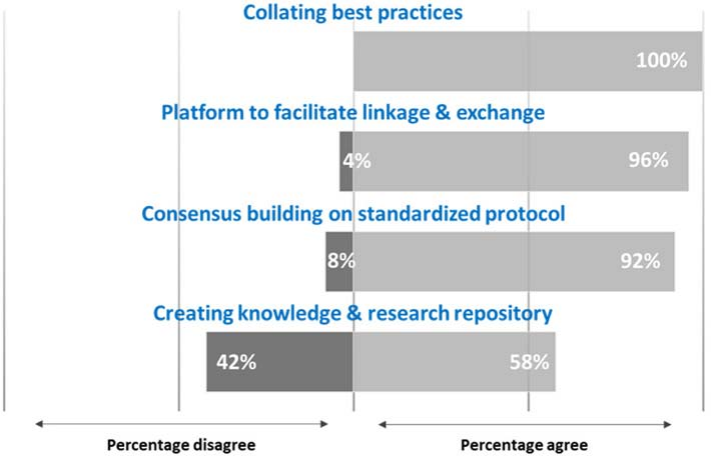
Network’s Role in implementation of tobacco control policies and program

Almost all the respondents agreed by the fact that research and practice network will help in implementation of tobacco control practices. Figure 1 depicts that almost all the respondents agreed on the fact that the research network will serve as a platform to facilitate linkage and information exchange and help create knowledge and research repository, collate best practices and build consensus on standardized protocols. Strong consensus was observed on the fact that the network will help in implementation of tobacco control policies and programs by sharing best practices and advocacy efforts.

#### Network structure and sustainability

Experts mentioned that the network should be multidisciplinary. A strong consensus was obtained on involvement of Government authorities, Non-Government Organizations and academia in the research and practice network. However, no consensus was observed on involvement of pharmaceutical and private sector in the network. In the first round of survey, experts stated that network can be sustained financially by securing funds from government (45) and international organizations (45 %). However, strong consensus was achieved only on fact that network can be sustained financially by Government funds.

#### Benefits of research and practice network in tobacco control

**Fig 2:**
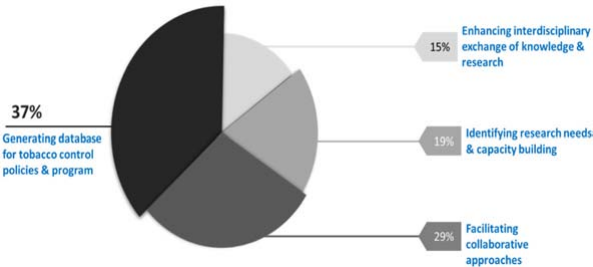
Benefits of research and practice network in tobacco control

In the first round of survey, almost all the respondents agreed by the fact that network will benefit researchers by creating a database for tobacco control policies and programs. Respondents also mentioned that the network will facilitate collaborative approaches and enhance capacity of researchers. Information sharing and capacity building of young researchers were the two important responses which achieved strong consensus by the experts (Fig. 2).

## Discussion and conclusions

This study produced the first national stakeholder-informed priority area for developing tobacco research and practice network in India. The consensus priorities highlight the most important and urgent needs in developing research and practice network in tobacco control. The expert panel approach was successful in building on stakeholder survey results to further define and prioritize research themes that reflected consensus. Final priorities were crafted into a statement which could be effectively communicated to the larger group of stakeholders.

The training, skills, and areas of expertise of researchers and practitioners working in tobacco control differ greatly [13]. For example, a researcher who studies the tobacco cessation uses different methods and requires a different skill set than a more applied researcher who compares the impact of different policies on tobacco control legislation. However, both types of research can influence the course of tobacco control. Thus, we captured responses of experts working in different areas in tobacco control on the focus of the research and practice network. The expert panel considered articulating research priorities that would be broad enough to encompass critical areas of research, yet sufficiently specific to guide the identification of actual priority areas for the research and practice network. Ultimately, the panel defined four major focus areas for the research and practice network identified in first round of survey i.e. Tobacco Cessation, legislation, diseases research and economics research. However, in the second round strong consensus was obtained only on the two areas i.e. tobacco cessation and economic research. Studies suggest that research on interventions to promote tobacco cessation and prevent tobacco initiation have been areas of relative neglect [14, 15]. Thus, research and practice network must accord priority to the design interventions to reduce tobacco consumption, promote cessation and prevent initiation.

WHO and the Global Tobacco Research Network have conducted studies assessing factors that foster and impede tobacco control research in low and middle income countries. These studies have shown consistently that funding, infrastructure, and resources are the most challenging impediments to building up tobacco control research [16, 17]. Similarly, findings of our study revealed that lack of funds, lack of resources, and lack of platform are the few factors which impedes research dissemination in tobacco control.

Consistent to the structure of tobacco research network in other developing and developed countries, majority of the respondents in our study emphasizes that research and practice network should be multidisciplinary and should involve researchers, academia, NGOs and Government bodies. However, there are examples of health promotion networks that purposefully excluded government representatives because of the perception that their presence would prevent the use of effective advocacy mechanisms [18]. Partnerships and alliances involving both government representatives and representatives of civil-society organizations thus pose challenges if the end result is perceived to be government action such as policy and program implementation.

Studies which assessed factors that impede tobacco control research in low and middle income countries have shown consistently that funding, infrastructure, resources are the most challenging impediments to building up tobacco control research [17–19]. Similarly, in our study experts from all professional affiliations chose funding issues as most important factor impeding formation of tobacco research network in both the rounds of survey. This underline the complexity of the issue and the need to increase and sustain public and private sector funding towards tobacco related research and formation of research network in India.

Analysis of the results of the Round I showed the researchers and practitioners stated that the research and practice network will benefit young researchers by improved information dissemination, technical support, access to tools and protocols, and increased career opportunities. Similar findings were reported in a study by Stillman et.al conducted in 2011 [13]. Considering the current and potential socio-economic burden of tobacco production and consumption there is an urgent need to bolster research capacity of young researchers in tobacco control.

Findings from this study revealed that Delphi method could be a valuable tool for building consensus on research and activities in tobacco control. The potential limitations of this study are low response rates in the online survey, low sample size in Round II of the survey, known weaknesses of the Delphi technique and the subjective process used for defining expert panelists for the sampling frame. The Delphi technique is also limited by whether the anonymous nature influences accountability and response rates [9]. Finally, the survey was concluded at the end of Round II even though full consensus was not achieved for all of the items. Although it is possible that greater consensus would have been reached with additional rounds, the decision was made to end the study due to concerns about limitation of time, panel fatigue and associated low response rates.

The present study envisaged that consensus-based research priorities would serve as a common rallying point for researchers and practitioners working in tobacco control to collaborate around common research goals. In this respect the study assesses perception of researchers and practitioners on available research infrastructure, capacities, organizational structure and themes of the research and practice network in tobacco control. The method of this study provides a model for the initial development of framework of tobacco research and practice network in India. Further work is needed to establish the validity of the responses. Consensus on the underlying constructs, themes, focus and constitution should be prioritized before setting up a research and practice network. These findings provide direction for future research based on the consensus views of renowned researchers, practitioners, policy makers and civil society members. It is acknowledged that priorities for research change as evidence builds to answer the questions. A much larger assessment which will collect information on organizational expertise and priorities and ongoing tobacco control research activities is needed.
